# Papillary thyroid carcinoma in graves’ disease: prevalence, clinicopathological features, and preoperative predictors

**DOI:** 10.1186/s12893-026-03602-y

**Published:** 2026-02-17

**Authors:** Tugba Matlim Ozel, Mine Yilmaz, Sezer Akbulut, Aykut Celik, Gorkem Yildiz, Nilsen Erdogan, Serkan Sari

**Affiliations:** 1 Department of General Surgery, Division of Endocrine Surgery, University of Health Sciences Turkey, Basaksehir Cam and Sakura City Hospital, Istanbul, Turkey; 2 Department of Pathology, University of Health Sciences Turkey, Basaksehir Cam and Sakura City Hospital, Istanbul, Turkey

**Keywords:** Papillary thyroid cancer, Graves disease, Thyroid-stimulating immunoglobulin (TSI), Thyroidectomy

## Abstract

**Background:**

The coexistence of Graves’ disease (GD) and papillary thyroid carcinoma (PTC) remains a subject of clinical debate. While PTC is frequently detected incidentally after thyroidectomy for GD, its prevalence, clinicopathological behavior, and preoperative predictors remain insufficiently defined.

**Methods:**

This single-center retrospective cohort study included 602 patients who underwent thyroidectomy between 2020 and 2025. Patients were categorized as: GD + PTC (*n* = 51); GD-only (*n* = 109); and PTC-only (*n* = 442). Demographic, biochemical, radiological, surgical, and pathological data were analyzed. Univariate and multivariate logistic regression models were used to identify factors associated with PTC development in GD.

**Results:**

The prevalence of PTC among GD patients was 31.9%. Compared with sporadic PTC, GD-associated tumors were smaller (median 5 mm vs. 12 mm, *p* < 0.001) and demonstrated fewer aggressive features including lymphatic invasion, capsular invasion, multifocality, bilaterality, and nodal metastasis (all *p* < 0.01). GD + PTC patients were younger and showed a lower female predominance than those with sporadic PTC. When compared with GD-only patients, the GD + PTC group had significantly lower thyroid-stimulating Immunoglobulin (TSI) titers (median 3.8 vs. 7.65 IU/L, *p* = 0.007) and a higher prevalence of ultrasound-detected thyroid nodules (64.7% vs. 27.5%, *p* < 0.001). In multivariate analysis, only US-detected nodules (OR 3.56, *p* = 0.003) and lower TSI levels (OR 0.95, *p* = 0.03) independently predicted PTC in GD.

**Conclusion:**

PTC is relatively common among surgically treated GD patients, yet presents predominantly as microcarcinoma with less aggressive histopathological features. The presence of ultrasound-detected thyroid nodules was the strongest preoperative predictor of malignancy. These findings support careful and systematic ultrasonographic assessment in patients with GD, with FNAB guided by established ultrasound risk patterns, nodule size thresholds, and high-risk clinical features, rather than indiscriminate lowering of biopsy thresholds.

## Introduction

Graves’ disease (GD) is an autoimmune thyroid disorder characterized by diffuse goiter, hyperthyroidism, and the presence of thyroid-stimulating immunoglobulins (TSI), leading to excessive stimulation and proliferation of thyrocytes [1, 2]. Graves’ disease represents the most common cause of hyperthyroidism worldwide and the disease shows a clear female predominance, affecting nearly 2% of women compared with 0.2% of men between the ages of 20 and 60 [3]. Although antithyroid drugs (ATDs) remain the initial therapeutic approach, a substantial proportion of patients experience treatment failure, relapse or side effects of ATDs eventually requiring definitive therapy with radioactive iodine ablation (RAI) or total thyroidectomy (TT) [4, 5]. Moreover, patients may be considered for TT under selected clinical circumstances, including suspected or concurrent malignancy, pregnancy planning, large goiter or compressive symptoms, moderate to severe Graves’ orbitopathy, intolerance or relapse on ATD therapy and RAI therapy, or when definitive therapy is preferred and radioactive iodine is undesirable. Surgical treatment offers rapid restoration of the euthyroid state and provides the advantage of identifying incidental thyroid cancer (TC), which would otherwise remain undetected in patients undergoing nonsurgical therapy [5, 6].

Papillary thyroid carcinoma (PTC) constitutes nearly 80% of all thyroid malignancies and represents the most frequent endocrine cancer globally, with its incidence continuing to rise [7, 8]. Historically, hyperthyroidism was believed to exert a protective effect against thyroid carcinogenesis due to suppressed thyroid-stimulating hormone (TSH) levels [9]. However, increasing evidence indicates that GD may coexist with and potentially predispose to TC, particularly PTC, with reported incidence rates ranging between 2% and 47% among surgically treated GD patients [10–14]. The proposed mechanisms include TSI-mediated proliferative signaling, angiogenesis, and enhanced tumor invasiveness [15]. Nevertheless, whether GD-associated PTC exhibits more aggressive behavior remains controversial, with recent studies reporting comparable oncological outcomes to euthyroid PTC [16, 17].

In addition to the coexistence of thyroid nodules, factors such as patient age, sex, TSI/TRAb titers, TSH dynamics, and other autoimmune indicators have been suggested to influence cancer risk in GD [11, 13, 17, 18]. Yet, literature data remain inconsistent, and there is currently no Graves’ disease–specific validated preoperative risk stratification model that reliably predicts malignancy beyond standard nodule-based assessment tools.In patients with Graves’ disease, diffuse autoimmune changes, increased vascularity, and pseudonodularity may complicate ultrasound interpretation, while the high prevalence of incidental microcarcinomas and selection bias inherent to surgical cohorts further limit the applicability of conventional risk stratification algorithms developed for euthyroid nodular disease [13, 17]. 

Therefore, the present study aims to evaluate the prevalence and clinicopathological characteristics of PTC detected in GD patients, compare oncologic outcomes between GD-associated PTC and sporadic PTC, and identify potential preoperative predictors of PTC among individuals diagnosed with GD. These findings may contribute to improved preoperative risk stratification and assist clinicians in tailoring surgical decision-making for patients with GD. 

## Materials-methods

### Study design and setting

This single-center study was conducted at the Department of General Surgery, University of Health Sciences, Basaksehir Cam and Sakura City Hospital, Division of Endocrine Surgery (Istanbul, Türkiye). Data were prospectively collected and retrospectively analyzed for patients operated between June 2020 and July 2025. The study protocol was approved by the Ethics Committee of Basaksehir Cam and Sakura Health Practices and Research Center (Approval No: KAEK/2025.12.463) and was performed in accordance with the Declaration of Helsinki. All patients provided preoperative written informed consent for participation in anonymous data collection for retrospective analyses. 

### Patient selection

The study included patients who underwent TT or TT with central neck dissection (TT + CND) for PTC and TT for GD. In PTC cases, CND was carried out based on clinical suspicion by US, typically on evidence of enlarged nodes, or confirmation of nodal disease during clinical work-up or intraoperatively. Only patients who underwent TT were included across all study groups. As TT represents the standard surgical approach for GD at our institution, this strategy ensured surgical uniformity and enabled reliable assessment of histopathological features such as multifocality, bilaterality, capsular invasion, and lymphovascular invasion, which cannot be accurately evaluated after lobectomy. Patients were categorized as follows:

Group 1: GD with coexisting PTC (GD + PTC).

Group 2: GD without malignancy (GD-only).

Group 3: PTC without GD (PTC-only).

**Fig. 1:**
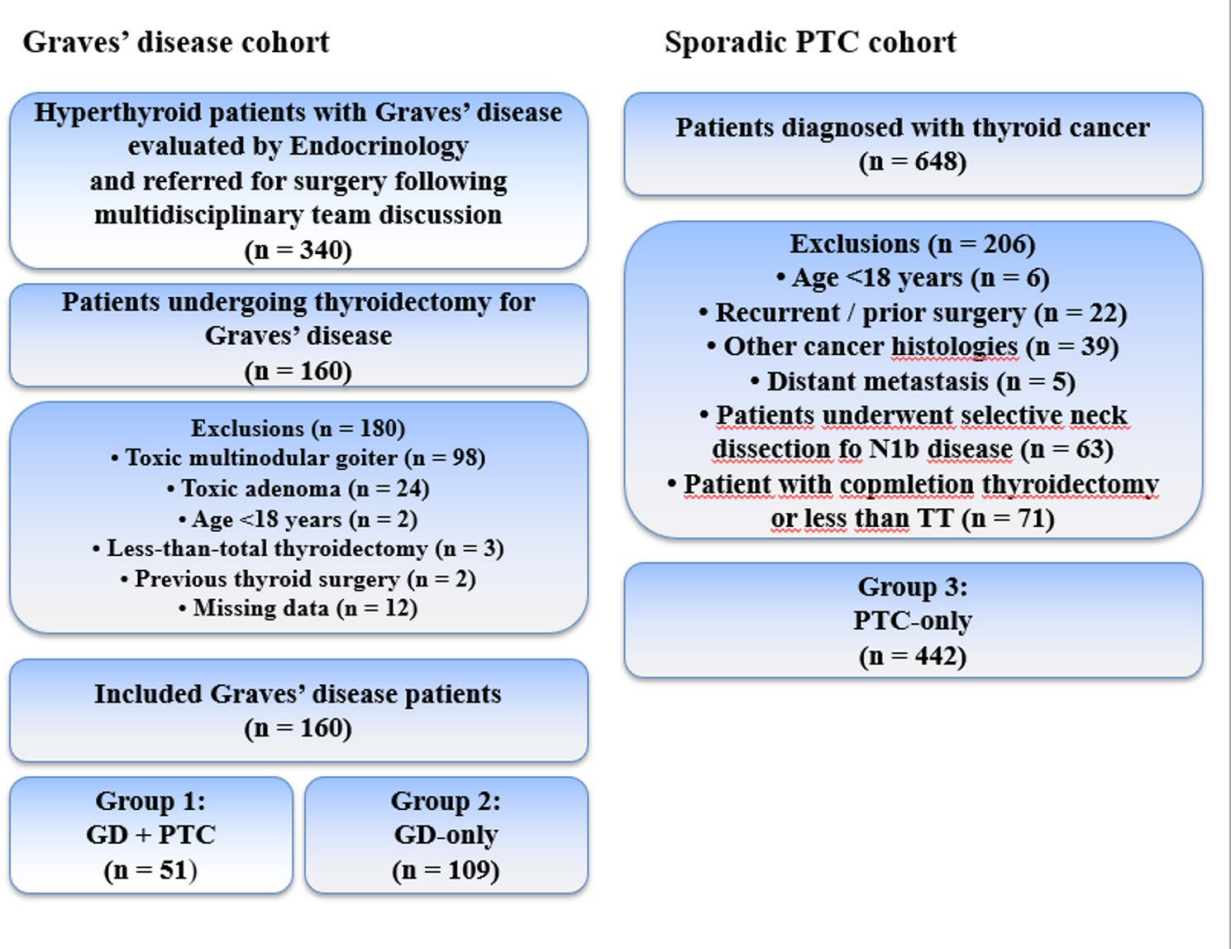
Flow diagram illustrating patient selection, exclusion criteria, and final study group allocation

The diagnosis of GD was established based on clinical manifestations of thyrotoxicosis, suppressed serum thyroid-stimulating hormone (TSH) levels, elevated free triiodothyronine (fT3) and/or free thyroxine (fT4) concentrations, positive TSI levels, and diffuse thyroid uptake on scintigraphy and/or characteristic ultrasonographic findings. PTCs with a diameter of 1 cm or less were defined as papillary microcarcinoma (PMC). Demographic data, TSI levels, autoimmune thyroiditis, medical therapy, nodule detection rates and histopathological results were compared between patients. Patients were excluded if they met any of the following criteria: surgery other than TT; hyperthyroidism due to toxic multinodular goiter or toxic adenoma; age < 18 years; prior thyroid surgery, history of head-and-neck malignancy; GD patients with non-papillary carcinoma histology; patients with any missing data. Patient selection, exclusion criteria, and group allocation are summarized in Fig. 1.

All patients underwent comprehensive preoperative evaluation including thyroid function tests (TSH, fT3, fT4), TSI, neck ultrasonography (US), and fine-needle aspiration biopsy (FNAB) when indicated. Serum TSI levels were measured using a chemiluminescence immunoassay (CLIA) on an automated analyzer (e.g., Siemens IMMULITE 2000 XPi / equivalent platform), with a reference range of < 0.1 U/L.TSI measurements were available for all included patients and were obtained at the time of initial diagnosis. Follow-up or post-treatment TSI levels were not included in the analysis. TRAb binding assays were not routinely performed at our institution and were therefore not included in the analysis. All US examinations were performed by a single experienced radiologist. Advanced ultrasound modalities such as elastography or contrast-enhanced ultrasound were not routinely available during the study period, and ultrasound reports were primarily structured according to based risk patterns. Cytological results were classified according to the 2023 Bethesda System for Reporting Thyroid Cytopathology.

### Surgical procedures

All operations were carried out by a team of five endocrine surgeons. In hyperthyroid patients, euthyroidism was achieved preoperatively with ATDs, β-blockers, and, when indicated, Lugol’s iodine solution administered for 7–10 days to reduce thyroid vascularity. The indication for surgery in GD was determined in multidisciplinary endocrine board meetings including endocrinologists, nuclear medicine specialists, radiologists, pathologists, and surgeons. Surgical indications included relapse or intolerance to medical therapy, failure or relapse after RAI treatment, large goiter or compressive symptoms, pregnancy planning, contraindication to RAI, suspicion of TC, indeterminate nodules, and/or patient preference. A history of prior RAI therapy was recorded for all patients. In cases with previous RAI exposure, the timing of FNAB relative to RAI treatment was documented.

### Histopathological evaluation

All surgical specimens were examined by the same pathology team using standardized histopathological and immunohistochemical techniques. PTC was diagnosed following the World Health Organization (WHO) classification and staged according to the 8th edition of the American Joint Committee on Cancer (AJCC) TNM staging system. Histopathologic features evaluated included tumor size, tumor type, multifocality, bilaterality, lymphatic, capsular and vascular invasion, extrathyroidal extension, lymph-node metastasis, and coexisting thyroiditis.

### Statistical analysis

Descriptive statistics were presented as mean ± standard deviation (SD) or median (25th–75th percentile) for continuous variables, and as frequency (n) and percentage (%) for categorical variables. Comparisons between clinical subgroups (PTC-only, GD + PTC, and GD only) were performed using the Student’s t-test or Mann–Whitney U test, as appropriate for the distribution of continuous variables. Variables found to be statistically significant or clinically relevant in univariate analyses were entered into a multivariate binary logistic regression model. Given the close clinical relationship between preoperative FNAB performance and thyroid nodule presence, FNAB was excluded from the multivariate model to avoid collinearity. Multivariate logistic regression was therefore performed using preoperative nodule presence and other clinically relevant variables. For each variable, odds ratios (ORs), 95% confidence intervals (CIs), and p-values were reported. Variables with OR values greater than 1 were interpreted as being positively associated with the dependent variable, whereas those with OR values less than 1 were interpreted as being negatively associated. All statistical analyses were performed using IBM SPSS Statistics for Windows, version 25.0 (IBM Corp., Armonk, NY, USA). A two-tailed p-value < 0.05 was considered statistically significant.

## Results

### Baseline Characteristics of the Study Population

A total of 602 patients were included in the final analysis, comprising 51 patients with Group 1, 109 patients with Group 2 and 442 patients with Group 3. The mean age of the entire cohort was 40.5 ± 11.5 years. In the overall study cohort (*n* = 602), 397 patients (65.9%) were female and 205 patients (34.1%) were male. Preoperative FNAB was performed in 82.7% of the overall study cohort. Among patients with GD, FNAB was performed selectively based on the presence of dominant or suspicious nodules and was required in 56 of 160 patients (35.0%). Thyroid nodules were detected on ultrasound in 39.4% of patients with GD.

Preoperative FNAB cytology, classified according to the Bethesda system, demonstrated the following distribution: BII in 7.0% (*n* = 35), BIII and BIV in 1.8% (*n* = 9), BV in 32.9% (*n* = 164), and BVI in 58.2% (*n* = 290) of patients. Regarding surgical procedures, TT was performed in 32.2% (*n* = 194), whereas TT+ unilateral/ bilateral CND was performed in 67.8% (*n* = 408). Autoimmune thyroiditis was present in 38.7% (*n* = 233) of patients and absent in 61.3% (*n* = 369). Fifteen patients in the Graves’ disease cohort had received prior RAI therapy. Preoperative FNAB was performed in three of these patients: two before RAI treatment with benign cytology results, and one after RAI therapy with a Bethesda V result. Baseline demographic and clinical characteristics are summarized in Table 1.

**Table 1:** Baseline demographic and clinical characteristics of study population (n:602)

Variables	Mean ± SD*	Median (Min-Max)
Age (year)	40.5 ± 11.5	40(18–69)
Variables	Number (*n*)	Percentage (%)
Gender distribution of the entire cohort (*n* = 602), (%) Female/ Male	397/205	65.9/34.1
Patient subgroup		
Group 1	51	8.5
Group 2	109	18.1
Group 3	442	73.4
Lymphocytic Thyroiditis		
No	369	61.3
Yes	233	38.7
Preoperative Nodule Situation in Group 1 and 2		
Absent	63	39.4
Present	97	60.6
Preoperative FNAB		
No	104	17.3
Yes	498	82.7
Preoperative Bethesda Category		
Bethesda II	35	7
Bethesda III and IV	9	1.8
Bethesda V	164	32.9
Bethesda VI	290	58.2
Surgical procedure		
TT**	194	32.2
TT + Unilateral/bilateral CND***	408	67.8

*SD: Standard Deviation, **TT: Total thyroidectomy, *** CND: Central neck dissection

### Comparison Between Sporadic PTC and Graves-Associated PTC (GD + PTC)

In our cohort, the overall prevalence of PTC among patients with GD was 31.9% (*n* = 51), and notably, 74.5% of these cancers (*n* = 38) were PMCs. Group 1 patients were significantly younger than those with PTC alone (40.2 ± 9.6 vs. 45.8 ± 12.5 years, *p* < 0.001). The female-to-male ratio was lower in the group 1 (33.3% vs. 78.5% females, *p* < 0.001). Preoperative TSH levels were significantly lower in Group 1 compared with Group 3 (median 0.07 vs. 1.76 mU/L, *p* < 0.001). Anti-TPO levels were slightly but significantly higher in Group 1 (median 13.9 vs. 11 IU/mL, *p* = 0.03). Fine-needle aspiration biopsy was performed in 47.1% of patients in Group 1 and in 100% of patients in Group 3 (*p* < 0.001). Cytology results were Bethesda II–IV in 54.2% of cases, whereas in Group 3 this rate was 0%. (Table 2)

**Table 2:** Comparison of demographic, clinical and histopathological findings between group 1 and group 3

Variables	Group 1 (*n* = 51)	Group 3 (*n* = 442)	*P* value
Age(year), *mean ± SD*	40.2 ± 9.6	45.8 ± 12.5	< 0.001*
Gender, *n (%)*			< 0.001‡
Female	17 (33.3)	347 (78.5)	
Male	34 (66.7)	95 (21.5)	
Preoperative TSH, median (Q1-Q3)	0.07(0.05–1.09)	1.76(1.1–2.44)	<0.001†
Preoperative Anti TPO, median (Q1-Q3)	13.9(9-126)	11(9-49.5)	0.03†
Postoperative adjuvant therapy (RAI) *n (%)*			< 0.001‡
No	48 (94.1)	158 (35.7)	
Yes	3 (5.9)	284 (64.3)	
Lymphocytic Thyroiditis *n (%)*			0.006‡
No	27 (52.9)	317 (71.7)	
Yes	24 (47.1)	125 (28.3)	
Preoperative FNAB *n (%)*			<0.001‡
No	27 (52.9)	0 (0)	
Yes	24 (47.1)	442 (100)	
Preoperative Bethesda category *n (%)*			< 0.001‡
Bethesda II	10 (41.7)	0	
Bethesda III and IV	3 (12.5)	0	
Bethesda V	6 (25)	157 (35.5)	
Bethesda VI	5 (20.8)	285 (65.5)	
Surgery type *n (%)*			<0.001‡
TT	43 (84.39)	42 (9.5)	
TT+unilateral/bilateral CND	8 (15.7)	400 (90.5)	
Tumor Diameter, median (Q1-Q3)	5 (3–11)	12 (8-18.25)	<0.001†
Tumor subtype, *n (%)*			0.17‡
Classic	32 (62.7)	240 (54.3)	
Follicular	8 (15.7)	47 (10.6)	
Tall cell	7 (13.7)	114 (25.8)	
Hobnail	1 (2)	4 (0.9)	
Others	3 (5.9)	37 (6.5)	
Lymphatic invasion, *n (%)*	22 (43.19)	335 (75.8)	<0.001‡
Multifocal disease, *n (%)*	17 (33.3)	247 (55.9)	0.002‡
Bilaterality, *n (%)*	12 (23.5)	239 (54.1)	< 0.001‡
Capsule invasion, *n (%)*			< 0.001‡
*Yes*	11 (21.6)	212 (48)	
*No*	15 (29.4)	32 (7.2)	
*Capsule absent*	25 (49)	198 (44.8)	
Extrathyroidal extension, *n (%)*	2 (3.9)	26 (5.9)	0.76‡
Vascular invasion, *n (%)*	4 (7.8)	42 (9.5)	0.81‡
Papillary Microcarcinoma *n (%)*	38 (74.5)	171 (38.7)	< 0.001‡
Lymph node metastasis, n (%)	8 (15.7)	227 (51.4)	< 0.001‡
Pathologic T stage, n (%)			< 0.001‡
T1a	38 (74.5)	171 (38.7)	
T1b	9 (17.6)	160 (36.2)	
T2	2 (3.9)	66 (14.9)	
T3	2 (3.9)	45 (10.2)	
Pathological N stage, n (%)**			0.023§
Nx	42 (9.5)	43 (84.3)	
N0	173 (39.1)	0 (0)	
N1a	227 (51.4)	8 (15.7)	
TNM Stage, n (%)**			1.000§
Not available	42 (9.5)	43 (84.3)	
EI	352 (79.5)	7 (13.7)	
EII	49 (11)	1 (2)	

*Student’s t test

*†Mann-Whitney U test*

*‡Chi square test*

*§Fisher exact (two-sided), p value < 0.05*

** For pathological N stage and TNM stage, p-values were calculated after exclusion of cases with unavailable nodal or stage information (Nx / not available)

## Tumor characteristics

GD + PTC cases demonstrated significantly smaller tumors (median 5 mm vs. 12 mm, *p* < 0.001), with PMC representing 74.5% of Group 1 compared with 38.7% of Group 3 cases (*p* < 0.001). Pathological T stage distribution differed significantly between the groups, with Group 1 demonstrating a higher proportion of T1a tumors (*p* < 0.001). After exclusion of cases with unavailable nodal information (Nx), pathological N stage also differed significantly between groups (*p* = 0.023). However, among patients with available staging data, AJCC/TNM stage distribution did not differ significantly between Group 1 and Group 3 (*p* = 1.000).

Aggressive features including lymphatic invasion (43.1% vs. 75.8%, *p* < 0.001), multifocality (33.3% vs. 55.9%, *p* = 0.002), bilaterality (23.5% vs. 54.1%, *p* < 0.001), capsule invasion (42.3% vs. 86.9%, *p* < 0.001), and lymph node metastasis (15.7% vs. 51.4%, *p* < 0.001) were significantly less common in Group 1. Total thyroidectomy alone was the predominant approach in Group 1 (84.3%), whereas in group 3 patients underwent TT + CND (90.5%) (*p* < 0.001). Postoperative RAI was required in only 5.9% of group 1 cases compared with 64.3% of group 3 cases (*p* < 0.001). (Table 2)

### Comparison Between GD + PTC and GD-only Patients

Baseline demographic and clinical variables were similar between GD + PTC and GD-only patients, including age (40.2 ± 9.6 vs. 40.5 ± 10.9 years, *p* = 0.85), BMI, medical therapy duration, and gender distribution (Table 3). Notably, Group1 patients had significantly lower TSI titers (median 3.8 vs. 7.65 IU/L, *p* = 0.007) and lower anti-TPO levels (median 13.9 vs. 119 IU/mL, *p* < 0.001). Thyroid nodules were more frequently detected in Group 1 than in Group 2 (64.7% vs. 27.5%, *p* < 0.001). Fine-Needle Aspiration Biopsy was more commonly performed in the group 1 (*p* = 0.03). Among the 160 patients who underwent TT for GD, preoperative FNAB was not performed in 104 patients (65.0%) due to the absence of dominant or suspicious nodules on US. The remaining 56 patients (35.0%) underwent preoperative FNAB. Cytology was classified as Bethesda category V or VI in 12 patients, prompting surgery with preoperative suspicion of malignancy. On final histopathological examination, PTC was confirmed in 11 of these 12 patients, while one patient with Bethesda V cytology had no malignancy. All remaining GD-associated PTCs were incidentally detected on postoperative histopathological examination.

**Table 3:** Comparison of demographic, clinical and histopathological findings between group 1 and group 2

Variables	Group 1 (*n* = 51)	Group 2 (*n* = 109)	*P* value
Age(year), *mean ± SD*	40.2 ± 9.6	40.5 ± 10.9	0.85*
Gender, *n (%)*			0.70‡
Female	17 (33.3)	33(30.3)	
Male	34 (66.7)	76(69.7)	0.70‡
Preoperative BMI**, mean ± SD	27.83 ± 5.61	26.62 ± 5.02	0.18*
Preoperative medical therapy period (month), mean ± SD	20.02 ± 5.13	21.31 ± 6.12	0.74*
Preoperative TSH, median (Q1-Q3)	0.07(0.05–1.09)	0.05(0.02–0.91)	0.36†
Preoperative Anti TPO, median (Q1-Q3)	13.9(9-126)	119(26.8–385)	< 0.001†
Preoperative TSI, median (Q1-Q3)	3.8(2.36–6.17)	7.65(2.45–21.6)	0.007†
Preoperative medical therapy type			
ATD	50(98)	108(99.1)	0.99‡
Steroid	17(33.3)	43(39.4)	0.46‡
RAI	3(5.9)	x12(11)	0.39‡
Preoperative FNAB *n (%)*			0.03‡
No	27 (52.9)	77(70.6)	
Yes	24 (47.1)	32(29.4)	
Preoperative Bethesda category *n (%)*			< 0.001‡
Bethesda II	10 (41.7)	25(78.1)	
Bethesda III and IV	3 (12.5)	6(18.8)	
Bethesda V	6 (25)	1(3.1)	
Bethesda VI	5 (20.8)	0(0)	
Preoperative Nodule Situation in Group 1 and 2			< 0.001‡
Absent	18 (35.3)	79 (72.5)	
Present	33 (64.7)	30 (27.5)	

*Student’s t test, †Mann-Whitney U test, ‡Chi square test, p value < 0.05

** BMI: Body mass index

**Table 4:** Univariate and multivariate analysis of predictors of PTC in GD

Variables	Univariate analysis			Multivariate analysis		
	OR*	95% CI**	*P*-value	OR	95% CI	*P*-value
Preoperative TSI	0.93	0.87–0.97	0.002	0.949	0.905–0.994	0.03
Preoperative anti TPO	0.997	0.995–0.999	0.006	0.998	0.996-1.000	0.11
Preoperative FNAB situation	2.14	1.08–4.25	0.03			
Preoperative nodule situation	4.83	2.37–9.84	< 0.001	3.83	1.76–8.29	0.001

* OR: Odds ration

**CI: Confidence Interval, p value < 0.05

### Predictors of Papillary Thyroid Carcinoma in Graves Disease

**Fig. 2:**
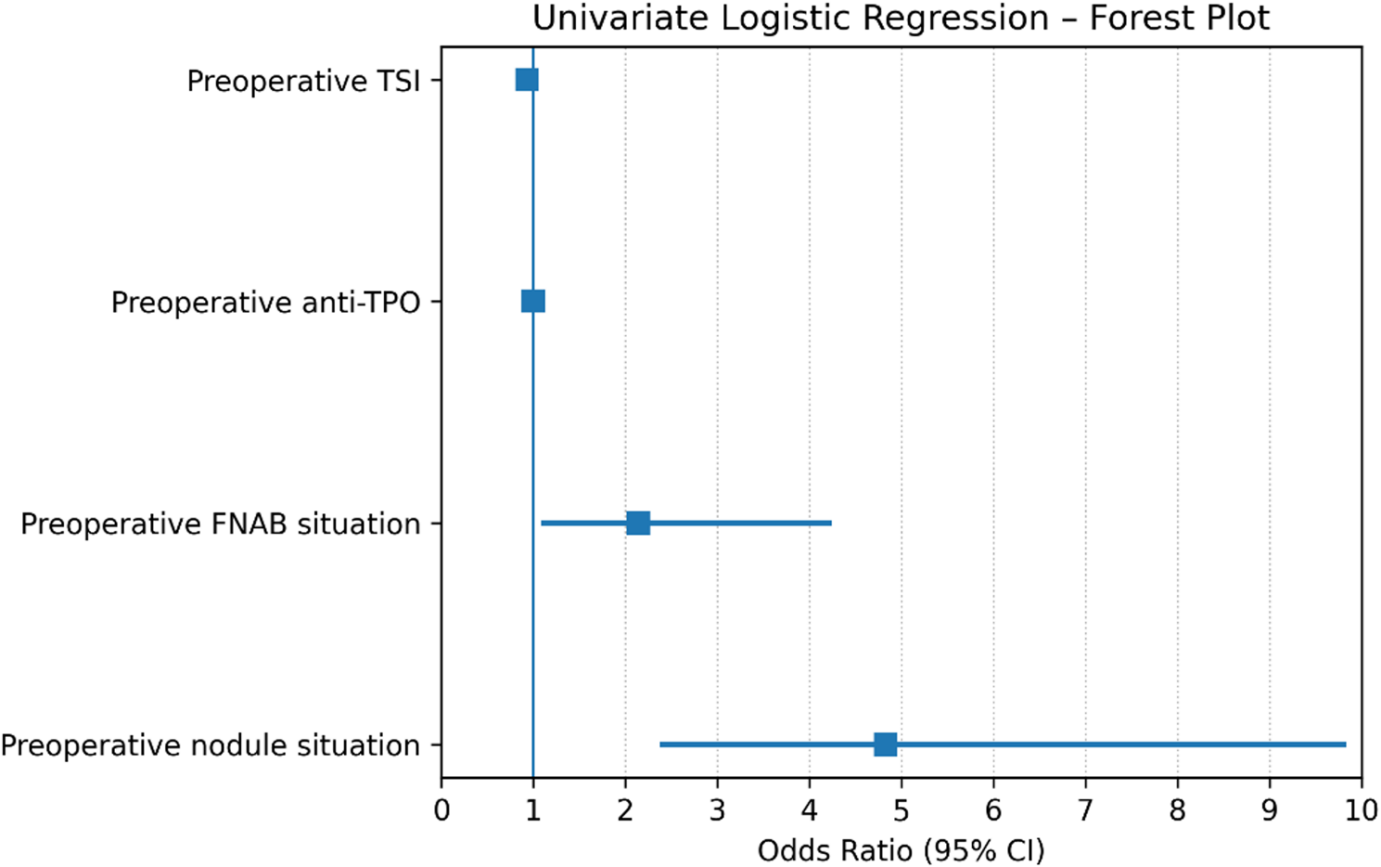
Forest plot of univariate logistic regression analysis

**Fig. 3:**
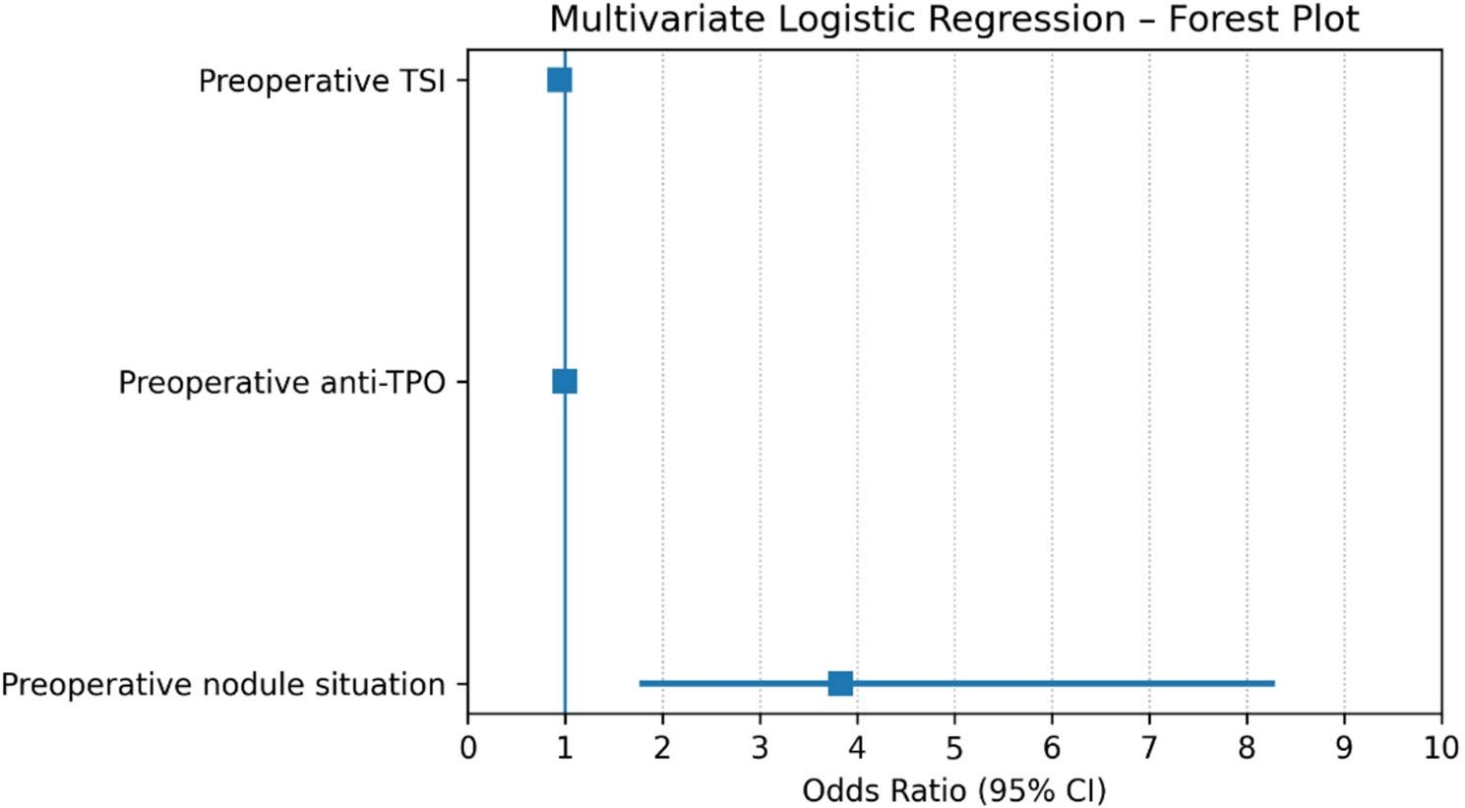
Forest plot of multivariate logistic regression analysis

In the univariate analysis, several factors were significantly associated with the presence of PTC in patients with GD, including lower preoperative TSI levels (OR = 0.93, *p* = 0.002), lower anti-TPO levels (OR = 0.997, *p* = 0.006), a history of preoperative FNAB (OR = 2.14, *p* = 0.03), and the presence of US-detected thyroid nodules (OR = 4.83, *p* < 0.001). In the multivariate model excluding FNAB, only two variables remained independently predictive: US detection of thyroid nodules (OR 3.83, 95% CI 1.76–8.29, *p* = 0.001), which was the strongest independent determinant, and lower TSI levels (OR 0.949, 95% CI 0.905–0.994, *p* = 0.03). Anti-TPO levels and FNAB history did not retain statistical significance in the multivariate analysis. The results of the univariate and multivariate logistic regression analyses are additionally summarized in forest plots to facilitate visual comparison of effect sizes and confidence intervals (Figs. 2 and 3).

## Discussion

The coexistence of GD and PTC has gained increasing attention as the overall incidence of thyroid pathology continues to rise. Although earlier hypotheses suggested that persistently suppressed TSH levels in hyperthyroidism might confer a protective effect against malignancy [19], more recent evidence indicates that GD and PTC commonly occur together [20]. Notably, contemporary cohorts report higher than expected rates of underlying TC in hyperthyroid patients, underscoring that the malignancy risk in GD is clinically relevant and warrants careful evaluation.

In our cohort, PTC was identified in 31.9% of surgically treated GD patients, with PMC accounting for nearly three-quarters of cases (74.5%). The prevalence observed in the present study falls within the higher range of rates reported in surgically treated GD cohorts and should be interpreted in the context of patient selection rather than as a population-level estimate. Consistent with our findings, Arosemena et al. reported PTC in 20% of 243 GD patients, 72% of which were microcarcinomas [12], whereas Moronta et al., analyzing 3,193 GD thyroidectomies and found incidental thyroid carcinoma in 12.7%, with 84.5% being T1 tumors [21]. Regional data from Guden et al. further support a higher malignancy risk in GD with nodules, demonstrating PTC in 27.8% of 212 patients, and 84.7% of cases were PMC [22]. Additionally, Keskin et al. reported TC in 28.1% (34/121) of GD patients undergoing thyroidectomy, with PTC as the predominant histologic subtype; notably, malignancy was significantly more common in nodular GD (38%) than in diffuse GD (16%) reinforcing the strong association between GD, nodules, and incidental malignancy [23]. In contrast, broader meta-analytic data report more modest prevalence estimates. Papanastasiou et al., combining 7 studies and 2,582 surgically treated GD patients, reported a pooled TC prevalence of 11.5% and found that the presence of at least one thyroid nodule increased cancer risk nearly fivefold [13]. Similarly, the systematic review by Jia et al. observed an incidental carcinoma prevalence of 7% in over 10,000 GD patients and concluded that GD itself did not confer a higher risk compared with other hyperthyroid etiologies [20]. An additional explanation for the wide variability in reported PTC prevalence across studies relates to differences in clinical practice patterns rather than intrinsic disease biology alone. Meta-analyses inevitably pool data from centers with heterogeneous diagnostic and surgical strategies, including variability in US surveillance intensity, FNAB thresholds, and indications for definitive surgical management in GD [13, 20]. In contrast, contemporary high-volume endocrine surgery series applying routine US, liberal FNAB use in nodular GD, and proactive surgical management consistently demonstrate higher detection rates of incidental PTC, predominantly microcarcinomas [12, 21–23]. From this perspective, our findings should not be viewed as a regional anomaly, but rather as an illustration of how diagnostic intensity and surgical decision-making directly shape observed PTC prevalence in GD. This practice-based framework helps reconcile discrepancies between meta-analytic estimates and modern surgical cohorts. These discrepancies likely reflect heterogeneity in iodine status, regional disease burden, surgical indications, and the sensitivity of preoperative US The relatively high prevalence of PTC observed in this study should be interpreted in light of a surgically selected cohort. In our series, 39.4% of GD patients had ultrasound-detected thyroid nodules, a feature known to influence referral for surgery and cancer detection. Importantly, surgical decision-making was not primarily driven by cytological suspicion of malignancy, as only a minority of patients underwent thyroidectomy due to Bethesda III–VI FNAB results. These findings suggest that while nodularity enriches the cohort for higher-risk features, the observed prevalence cannot be explained solely by malignancy-oriented surgical selection.

Overall, while meta-analyses suggest only a modest increase in risk, contemporary surgical series particularly from endemic regions demonstrate substantially higher PTC rates, emphasizing the need for careful ultrasonographic evaluation and liberal use of FNAB in GD patients presenting with nodules. Although several of our findings are consistent with prior literature, the present study contributes by integrating ultrasonographic, immunological, and surgical–pathological data into a clinically actionable risk stratification framework [10–14]. Rather than challenging existing guidelines, the present study provides real-world, institution-level data that support and operationalize current recommendations. By integrating US findings with immunological parameters, our results contribute to a more individualized application of guideline-based surveillance strategies, particularly in surgically treated GD patients. This approach may help avoid both underdiagnosis in nodular disease and unnecessary aggressive investigation in non-nodular, autoimmune-dominant cases. While thyroid nodularity has been repeatedly identified as a key risk factor for malignancy in GD, our results demonstrate that lower TSI levels independently predict PTC, whereas markers of autoimmune activity and hyperthyroid severity do not. These findings suggest that, within surgically treated GD cohorts, malignancy detection is more closely associated with structural disease expression—particularly the presence of discrete thyroid nodules—than with the absolute magnitude of circulating autoimmune antibody titers alone. Importantly, structural thyroid changes in GD are themselves closely intertwined with the underlying autoimmune process, treatment-related heterogeneity (ATD or RAI), and differences in surveillance intensity; therefore, this association should be interpreted cautiously, and causality should not be inferred. Potential contributors include lead-time bias related to more intensive ultrasonographic surveillance, variations in surgical referral patterns, and treatment-related modulation of antibody levels over time. Moreover, diffuse hypervascularity and pseudonodularity characteristic of Graves’ disease may complicate ultrasound interpretation, thereby limiting the direct transferability of malignancy risk stratification tools developed for euthyroid nodular disease and reinforcing the need for risk-pattern–based, size-threshold–aligned FNAB decisions in selected GD patients. Moreover, by directly comparing GD-associated and sporadic PTC treated under identical institutional standards, we confirm that GD-associated tumors are predominantly indolent despite their relatively high prevalence [15–18]. Collectively, these findings support a selective surveillance strategy in GD, emphasizing careful US evaluation and targeted FNAB in nodular disease rather than routine aggressive investigation of all patients.

In our cohort, GD-associated PTC appeared at a significantly younger age and—with an unexpectedly higher proportion of male patients—showed a demographic pattern that differs from classic sporadic PTC. While this may suggest that the autoimmune and hyperthyroid environment of GD modifies the usual demographic profile of PTC, alternative explanations must be considered. Arosemena et al. similarly reported that PTC in GD tended to present earlier and with a less pronounced female dominance, most tumors being microcarcinomas with indolent features [12]. In contrast, larger meta-analyses by Mekraksakit et al. and Staniforth et al. did not identify consistent age or sex differences between GD-associated and non-GD PTC, indicating substantial heterogeneity in published data [24, 25]. Alternatively, differences in patient selection and surgical referral patterns may contribute to this observation, as male GD patients are more likely to present with severe or symptomatic disease requiring surgical management [12, 21]. Accordingly, surgically treated GD cohorts tend to include a higher proportion of younger and male patients compared with the overall GD population, independent of malignancy-related factors [21, 24]. This consideration underscores that the observed demographic differences should be interpreted cautiously and viewed as hypothesis-generating rather than causal.

Although GD-associated PTC was more frequently diagnosed at an earlier pathological T stage, overall AJCC/TNM stage did not differ significantly among patients with available staging data. This finding suggests that differences in tumor size and nodal involvement may partly reflect surveillance intensity and extent of nodal evaluation rather than uniformly earlier disease stage. Whether PTC developing in GD behaves more aggressively than classical PTC remains controversial. Some earlier and mid-period studies suggested higher rates of multifocality, extrathyroidal extension, and nodal metastasis in GD-associated tumors, with certain reports emphasizing the higher prevalence of tall-cell variant in GD [26, 27]. These observations supported the hypothesis that TSI-mediated stimulation may promote more aggressive tumor biology. Consistent with our findings, Arosemena et al. reported that 72% of PTCs in GD were microcarcinomas and predominantly stage I, suggesting a largely indolent course [12]. Similarly, Moronta et al., analyzing over 3,000 GD thyroidectomies, found 84.5% of incidental cancers were T1 lesions with low rates of nodal disease [21]. More recent large dataset analyses have further suggested that although carcinoma is more frequently encountered in GD, it tends to be less aggressive histologically and clinically [22, 28]. Our results align more closely with this recent body of evidence. GD-associated PTC cases exhibited significantly reduced rates of lymphatic invasion, capsule invasion, multifocality, bilaterality, and lymph node metastasis compared with euthyroid PTC. These findings strongly support the concept that the GD-associated PTC phenotype is biologically less aggressive, despite its increased incidence. Overall, when interpreted alongside the broader literature, our findings suggest that while the frequency of PTC in GD may be elevated particularly in endemic, nodular populations the biological behavior of these tumors is typically more indolent than PTC occurring in euthyroid patients. This distinction is clinically relevant: it supports active and systematic cancer screening in GD, while also cautioning against overtreatment solely based on the coexistence of GD.

In our cohort, US-detected thyroid nodules and lower TSI levels were the only independent predictors of PTC in patients with GD, highlighting structural thyroid abnormalities and altered antibody dynamics as key markers of malignancy risk. Preoperative FNAB did not retain independent significance in the multivariate model once nodule presence was accounted for, reflecting its role as a downstream diagnostic consequence of nodule detection rather than an independent biological risk factor. The observed differences in anti-TPO levels across the three study groups likely reflect variations in autoimmune disease phenotype rather than a direct association with malignant transformation. Anti-TPO antibodies primarily serve as markers of thyroid autoimmunity and lymphocytic inflammation and are known to be elevated in diffuse autoimmune thyroiditis, including GD and Hashimoto’s thyroiditis, independent of nodular architecture or oncologic risk [29, 30]. Several studies have demonstrated that GD patients with a predominantly diffuse autoimmune phenotype tend to exhibit higher anti-TPO titers, whereas those with nodular or structurally remodeled glands often show lower antibody levels despite active disease. This distinction may partly explain the lower anti-TPO levels observed in GD patients with concomitant PTC, in whom nodularity and architectural alteration rather than diffuse lymphocytic infiltration appear to dominate the disease phenotype. Importantly, existing evidence does not support a direct oncogenic role for anti-TPO antibodies. Large retrospective series and meta-analyses have failed to demonstrate a consistent association between anti-TPO positivity or titer magnitude and TC risk [30, 31]. Instead, anti-TPO levels seem to reflect the underlying immune milieu and inflammatory burden of the thyroid gland rather than malignant potential. Accordingly, the differences in anti-TPO levels observed in our cohort should be interpreted as markers of heterogeneous autoimmune expression across GD phenotypes rather than independent predictors of papillary thyroid carcinoma. This interpretation aligns with our broader findings that structural thyroid abnormalities, particularly nodularity, are more robust indicators of malignancy risk than the intensity of humoral autoimmunity.

Nodularity has consistently emerged as the strongest and most reproducible predictor of cancer in GD across contemporary high-volume studies. Guden et al., in a large cohort comparing euthyroid nodular goiter–associated DTC with GD-associated DTC, demonstrated that GD patients with nodules had a significantly higher prevalence of carcinoma, and nodularity was the primary differentiating feature associated with malignancy and tumor multifocality [22]. This finding is reinforced by Soares et al., who reported nodules in 53% of patients and showed that nodule number and size, rather than antibody titers, were associated with cancer risk [32]. Ren et al. found thyroid nodules in 22.7% of 423 GD patients, with cancer in 13.7% of surgically treated cases, and demonstrated that nodules—particularly those > 1 cm—were the main predictors of incidental DTC, whereas TSI titers were not predictive [33]. Meta-analytic evidence aligns with these conclusions: Umbrella review by Palella et al. graded as “strong” the evidence linking nodules in GD with increased TC risk, with pooled ORs consistently above 5.0 [26]. The comparison between GD patients with and without concomitant PTC should be interpreted with caution, as surgical selection inherently influences this analysis. Nodule presence functions both as a clinical risk marker and as a factor contributing to surgical referral, introducing a degree of conceptual overlap. Importantly, however, surgery in the GD + PTC group was not predominantly driven by suspicious cytology, as only a minority of patients underwent thyroidectomy due to Bethesda III–VI FNAB results, while most were operated on for conventional GD-related indications such as refractory disease, medication intolerance, ophthalmopathy, or compressive symptoms. Although nodularity differentiates GD patients with and without PTC, this association should be regarded as descriptive and hypothesis-generating rather than causal. Neverthless in the context of Graves’ disease, where diffuse autoimmune changes and pseudonodularity are common, the identification of a true discrete thyroid nodule on ultrasound remains clinically meaningful and should not be dismissed as a nonspecific finding. Our results highlight that nodularity retains its dominant predictive value for malignancy even within this complex autoimmune background.

Conversely, the role of thyroid-stimulating antibodies as predictors remains uncertain. The review by Damaskos et al., evaluating three retrospective series totaling 916 GD patients, concluded that TSI levels were not significantly correlated with the presence of incidental thyroid carcinoma [34]. Finnerty et al. likewise found that preoperative TSI was not associated with aggressive features or recurrence in concomitant GD-PTC [35]. Several biologically and clinically plausible mechanisms may explain the observed inverse association between TSI levels and concomitant PTC in surgically treated GD patients. First, TSI may act less as an upstream oncogenic driver and more as a marker of GD phenotype. Patients with lower stimulating activity often exhibit a more structural or nodular disease pattern, and thyroid nodularity is the most consistently reported upstream correlate of malignancy risk in GD cohorts [26, 32]. In this context, cancer risk appears to be driven primarily by architectural remodeling rather than the intensity of circulating stimulating antibodies. Second, TSI levels are dynamic and influenced by disease duration and treatment; antithyroid drug therapy and longer disease course are associated with declining antibody titers, whereas radioiodine therapy may cause transient increases. Thus, lower preoperative TSI may reflect disease phase or prior treatment exposure rather than a direct biological driver of tumor initiation [36–38]. Finally, circulating TSI levels may not accurately reflect intrathyroidal receptor stimulation in tumor-bearing glands, as altered TSH receptor expression and local immune–tumor interactions can decouple serum antibody levels from relevant intrathyroidal signaling [39]. Given the dynamic nature of TSI and its modulation by disease duration and antithyroid therapy, the observed association between lower TSI levels and concomitant PTC should be interpreted cautiously and regarded as hypothesis-generating rather than evidence of a direct biological effect.

### Study limitations

This study has several limitations that should be acknowledged. First, its retrospective, single-center design and inclusion of surgically treated GD patients introduce selection bias; therefore, the observed prevalence of papillary thyroid carcinoma reflects a surgical cohort and may not be fully representative of the broader GD population managed medically or with RAI. The small number of patients with prior RAI exposure further limits formal assessment of RAI-related cytologic effects. In addition, regional iodine status and background nodular thyroid disease may influence incidental microcarcinoma detection and generalizability. Second, although biochemical and imaging data were prospectively collected, antibody measurements were performed using a single laboratory platform, which may limit external validity, and the identified predictive associations should not be extrapolated to non-operatively managed GD populations. Third, conclusions regarding a “less aggressive” GD-associated PTC phenotype are based on histopathological features, as long-term oncologic outcomes could not be reliably assessed due to limited follow-up and the predominance of microcarcinomas. Additionally, the lack of molecular profiling restricts insights into whether GD-associated tumors harbor distinct oncogenic mutations compared with euthyroid PTC. Finally, ultrasonographic detection of nodules—an independent predictor in this study—was performed by a single radiologist, which, although ensuring internal consistency, may limit reproducibility in broader clinical practice settings. During the study period, a large proportion of patients were operated on according to an institutional protocol that included prophylactic central neck dissection in clinically node-negative PTC. While this strategy may increase the detection of occult nodal disease, it was not selectively applied based on tumor aggressiveness and was uniformly implemented according to institutional policy. Differences in the extent of nodal evaluation and the high proportion of cases with unavailable nodal or AJCC/TNM staging, particularly in the GD-associated PTC group, may have influenced pathological N classification and stage-based comparisons.

## Conclusion

In this surgically treated cohort, PTC was frequently identified in patients with GD, most commonly as PMC. GD-associated PTC demonstrated distinct clinicopathological features compared with sporadic PTC, including smaller tumor size and lower rates of aggressive histopathological characteristics. These findings are based on histopathological evaluation and should not be interpreted as evidence of long-term oncologic outcomes. Preoperative ultrasonography-detected thyroid nodules and lower TSI levels emerged as independent predictors of malignancy in GD patients, highlighting the importance of careful structural assessment within the autoimmune thyroid background. These findings support a risk-stratified diagnostic approach in GD, emphasizing ultrasound-guided evaluation and selective FNAB based on established sonographic risk patterns rather than routine aggressive investigation. Further prospective studies incorporating longitudinal outcomes are warranted to clarify the prognostic implications of PTC detected in the setting of GD. 

## Data Availability

The raw data supporting the conclusions of this article will be made available by the authors without undue reservation.

## Abbreviations

GD: Graves’ Disease
PTC: Papillary Thyroid Carcinoma
PMC: Papillary Microcarcinoma
TSI: Thyroid-Stimulating Immunoglobulin
TRAb: Thyroid-Stimulating Hormone Receptor Antibody
US: Ultrasound
FNAB: Fine-Needle Aspiration Biopsy
TT: Thyroid Cancer
TT: Total Thyroidectomy
CND: Central Neck Dissection
ATD: Antithyroid Drug
RAI: Radioactive Iodine Therapy
AJCC: American Joint Committee on Cancer
